# Use of Multiple EEG Features and Artificial Neural Network to Monitor the Depth of Anesthesia

**DOI:** 10.3390/s19112499

**Published:** 2019-05-31

**Authors:** Yue Gu, Zhenhu Liang, Satoshi Hagihira

**Affiliations:** 1 Key Laboratory of Computer Vision and System (Ministry of Education), School of Computer Science and Engineering, Tianjin University of Technology, Tianjin 300384, China; 2 Institute of Electrical Engineering, Yanshan University, Qinhuangdao 066004, China; zhl@ysu.edu.cn; 3 Department of Anesthesiology, Graduate School of Medicine, Osaka University, Osaka 565-0871, Japan; hagihira@anes.med.osaka-u.ac.jp

**Keywords:** depth of anesthesia, electroencephalogram, bispectral index, artificial neural network

## Abstract

The electroencephalogram (EEG) can reflect brain activity and contains abundant information of different anesthetic states of the brain. It has been widely used for monitoring depth of anesthesia (DoA). In this study, we propose a method that combines multiple EEG-based features with artificial neural network (ANN) to assess the DoA. Multiple EEG-based features can express the states of the brain more comprehensively during anesthesia. First, four parameters including permutation entropy, 95% spectral edge frequency, BetaRatio and SynchFastSlow were extracted from the EEG signal. Then, the four parameters were set as the inputs to an ANN which used bispectral index (BIS) as the reference output. 16 patient datasets during propofol anesthesia were used to evaluate this method. The results indicated that the accuracies of detecting each state were 86.4% (awake), 73.6% (light anesthesia), 84.4% (general anesthesia), and 14% (deep anesthesia). The correlation coefficient between BIS and the index of this method was 0.892 (p<0.001). The results showed that the proposed method could well distinguish between awake and other anesthesia states. This method is promising and feasible for a monitoring system to assess the DoA.

## 1. Introduction

During surgery, general anesthesia is necessary and important to ensure the safety of patients. Overdose anesthesia may make the recovery time longer, while inadequate anesthesia may lead to intraoperative awareness and psychological effects on patients [1]. However, there is not an identical definition of the anesthetic state among anesthesiologists [2]. Objective, noninvasive and reliable monitoring depth of anesthesia (DoA) is still a clinical concern for anesthesiologists [3].

Many techniques and clinical indices such as blood pressure and heart rate have been used to indicate DoA. However, there are some drawbacks among these methods so that they are unreliable for assessing DoA. For example, the analysis result may be different depending on the types of surgery and drugs [4]. Due to the central nervous system (CNS) affected by the anesthetic drugs, the electroencephalogram (EEG) originating in CNS has been focused on by researchers [5]. The EEG reflects the brain activities and contains lots of information about anesthesia, so it has been widely used to assess DoA [6,7,8].

In recent decades, numerous EEG-based methods have been proposed to develop an index to assess the anesthetic drug effects during general anesthesia, such as narcotrend index (NI), BetaRatio (BR), 95% spectral edge frequency (SEF95), SynchFastSlow (SFS), median power frequency (MPF), high-order spectral analysis and entropy etc. [9,10,11]. Based on these methods, some commercial monitors of DoA such as BIS (Aspect Medical Systems, Newton, MA) [12], index of consciousness (IoC) (Morpheus Medical, Barcelona, Spain) [13], and M-entropy (GE Healthcare, Helsinki, Finland) [14] have been developed. Among these monitors, the BIS monitor is a popularly used device to estimate the DoA [15], in which several parameters derived from the EEG signals with different weights are combined using a nonlinear function to produce a dimensionless numerical index [16]. The BIS index ranges from 0 to 100 and different numbers of BIS represents different anesthesia states (80–100: awake; 60–80: light anesthesia; 40–60: general anesthesia; under 40: deep anesthesia). The BIS monitor is an important assistant equipment in clinical care, however, the calculation of BIS index is still unclear, with some researchers even finding that BetaRatio was positively correlated with BIS at BIS more than 60. SynchFastSlow and SEF95 were positively correlated with BIS at a BIS range of 30 to 80 [17].

As we know, including the brain, many dynamic systems exhibit strong nonlinearity [18,19,20]. Therefore, nonlinear analysis methods may be better in anesthesia study [21]. Permutation entropy (PE) as a typical nonlinear analysis method has been developed to measure the complexity of EEG signals during anesthesia and coma [22,23,24,25]. It is derived from complexity measure of symbolic dynamics [26]. According to Liang’s study, PE performs better than other entropy methods in several aspects [7]. For the conceptually simple, computationally efficient and artifact-resistant, PE is suitable for assessing DoA. However, it has a poor performance during the burst-suppression period, because of the characteristic of high-frequency waves [7].

Due to the complex changes of the EEG during different anesthetic states, none of the EEG-based features can assess the DoA completely and reasonably all the time [27]. Therefore, it is necessary to use multiple features to track the consciousness transition from awake to deep anesthesia [28]. Some studies have been done recently. Ortolani used an artificial neural network (ANN) to integrate 13 EEG features to assess DoA [29]. In Guo’s study, the wavelet transform method was used to analyze the anesthesia monitoring EEG signals, and the extracted features were clustered by wavelet classifier to estimate DoA [30]. Liu et al. used random forest with nonstationary signal features to estimate DoA through human EEG signal at different levels of unconsciousness [31]. Shalbaf et al. assessed DoA using Adaptive Neurofuzzy System with spectral, fractal, and entropy [32]. Then they assessed the level of anesthesia with sevoflurane in 17 patients using support vector machine (SVM) with Shannon entropy and frequency features [33]. Shalbaf used an ANN to integrate 2 entropy features to assess DoA [4]. Jiang et al. showed that ANN was one of the artificial intelligent methods that could provide the most accurate results through matching the trained model [2]. These motivate us to explore whether or not an ANN, which is based on multiple features, including frequency domain and nonlinear features, could be used to assess DoA.

In this paper, we apply the indices of PE, SEF95, BR, and SFS as the inputs of ANN to estimate the different anesthetic states. BIS values were used as reference output. The performance of this method was evaluated by sensitivity and classification accuracy as in Shalbaf’s study [4]. In addition, we compared the performance of ANN with another popular machine-learning algorithm, SVM.

## 2. Materials and Methods

### 2.1. Subjects and EEG Recordings

The EEG signals were recorded from 16 adult patients (25–63 years old) under general anesthesia using the ASPECT A-1050 monitor (Aspect Medical Systems, Natick, MA, USA). After cleaning the skin, the electrodes of BIS were applied to obtain the EEG signals over the forehead of all patients. The EEG montage was Fpz-At1, and the reference lead was placed at Fp1 [21]. Raw EEG data was sampled by 128 Hz. This study was approved by the ethics committee of Osaka Prefectural Habikino Hospital. Written informed consent was obtained from each patient.

The patients were anesthetized with propofol. The target effect-site concentration of propofol was 3.5 micro mg/mL. Anesthesia was maintained using a target-controlled infusion (TCI) system. An intervention was made by ketamine i.v. etc. during the time course of propofol anesthesia. The data analysis was performed on the MATLAB (version 8.2, MathWorks Inc.) software.

### 2.2. EEG Processing

**Figure 1:**
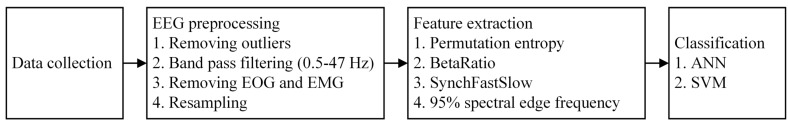
The flow chart of EEG processing.

Eye movements, muscle activities, and industrial frequency noise are the main artifacts in EEG recordings. These artifacts make the analysis results of the DoA unconvincing, especially during the awake state [6]. Therefore, all the EEG signals were preprocessed before subsequent analysis. First, outliers which were detected by a threshold determined by mean and standard deviation were removed [34]. Second, we used a band pass (0.5–47 Hz) finite impulse response (FIR) filter to remove baseline drift and industrial frequency noise. FIR filter does not disturb the phase information of the EEG signals. Third, the stationary wavelet transform with an optimal threshold was used to remove the electrooculogram (EOG) artifacts [35]. An inverse filter was then used to detect and remove electromyogram (EMG) artifacts and other transient high-amplitude artifacts. Fourth, the EEG data was resampled to 100 Hz. Finally, 1-min epochs were extracted from the artifact-free EEG. The information of the EEG data is listed in Table 1. The unit of raw EEG length and preprocessed EEG length is in minutes. The number of samples means how many samples each patient can provide to train or test the classifier. In this paper, we used three kinds of features as the inputs of ANN to assess the DoA. They are frequency domain and entropy features, respectively. We calculated BR, SEF95, and SFS as the frequency-domain features, and PE as the entropy feature. The EEG processing flow is shown in Figure 1. Additionally, considering that ANN might work better with raw signals as input, because it can learn the distinct feature of a particular class, we also used the preprocessed EEG as input to train the ANN. The corresponding results were included in the Appendix A.

### 2.3. Permutation Entropy Algorithm

PE that gives a quantitative complexity measure of a dynamical time series was originally proposed by Bandt and Pompe [26] and has been successfully used to analyze EEG series of anesthesia.

Given an *N*-point time series X={x(1),x(2),…,x(N)}, vectors $X_{i}=\left\{x(i),x(i+\tau ),\ldots ,x(i+(m-1)\tau )\right\}$, 1≤i≤N−(m−1) with the embedding dimension *m* and lag τ are constructed. $X_{i}$ can be then rearranged in a decreasing order. There will be K=m! possible order patterns for *m* dimensions, which are also known as permutations. Each vector $X_{i}$ can be represented by one of the *K* permutations. $P_{j}$ represents the probability of the *j*th permutation occurring. Then, the normalized PE is expressed as:

(1) $$PE=\frac{-\sum_{j=1}^{K}P_{j}\ln P_{j}}{\ln K}$$

The range of PE value is zero to one. The smaller the PE value is, the more regular the time series is, and vice versa. The calculation of PE depends on the selection of the data length *N*, embedding dimension *m*, and lag τ. According to the suggestions provided by previous studies, the data length *N* and lag τ are set to 1000 and 1, respectively [6]. The appropriate embedding dimension *m* is related to the signal and its sampling frequency. In this study, different parameter *m* values will be tested (m=3 to 6).

### 2.4. Frequency-Domain Algorithm

As mentioned above, in this study we used three frequency-domain features: BR, SFS, and SEF95. As described by Rampil [16], BR is the log ratio of the spectral power in 30–47 Hz band and 11–20 Hz band. The formula is described as follows:

(2) $$BR=\log\frac{SP(30-47\;\mathrm{Hz})}{SP(11-20\;\mathrm{Hz})}$$

where SP represents the spectral power in some frequency bands.

SFS is the log ratio of the sum of bispectral power in 0.5–47 Hz and 40–47 Hz. The formula is described as follows:

(3) $$SFS=\log\frac{BISP(0.5-47\;\mathrm{Hz})}{BISP(40-47\;\mathrm{Hz})}$$

where BISP represents the sum of bispectral power in some frequency bands.

SEF95 is the frequency below which 95% of the spectral power exists. According to previous study, SEF decreases during general anesthesia with isoflurane or propofol compared with the awake state [36].

### 2.5. Artificial Neural Network

The ANN is a flexible, nonparametric, parallel computing model which was developed based on the presumed nerve structure of the human brain [37]. The ANN is usually made up of many interconnected nodes in multiple layers, which are input layer, hidden layer, and output layer, respectively. It is the so-called multilayer perceptron which is the most commonly used ANN structure. All the nodes and layers are arranged in a feedforward manner. Each node in the input layer receives external information. Each node in the output layer produces the model solution and outputs a final result. Between input layer and output layer, there are usually one or more hidden layers which identify the complex patterns in the data [38]. To achieve the best output, ANN repeats constant learning and error correction. The ANN is a humanlike system that can understand new problems, analyze them, and finally sum up the best results.

There are usually two learning rules in ANN: supervised learning and unsupervised learning. In this study, we perform the back-propagation algorithm, which is one of the supervised and the most commonly used learning algorithms.

### 2.6. Support Vector Machine

SVM is a popular machine-learning approach which was first introduced by Vapnik and his colleagues [39,40]. SVM can not only be applied to classification problems, but also to regression problems when the response variable is a real-valued number, resulting in support vector regression (SVR) [41]. Suppose we have a set of data including *N* predictor variables and observed response values, $D=\{\left(x_{1},y_{1}\right),\ldots ,\left(x_{N},y_{N}\right)\}$. The goal is to find a function f(x) which deviates from the observed response values by a value less than ε for each predictor variable.

To find the linear function,

(4) f(x)=wx+b

The optimal regression function is given by the minimum of the function,

(5) $$min\frac{1}{2}{∥w∥}^{2}+C\sum_{n=1}^{N}\left(\xi_{n}^{-}+\xi_{n}^{+}\right)$$

where *C* is a prespecified value which controls the tradeoff between the close fit to the data and regularization, $\xi_{n}^{-}$ and $\xi_{n}^{+}$ are slack variables representing upper and lower constraints on the outputs of the system.

The optimization problem mentioned above is to solve in its Lagrange dual formulation. To obtain the dual formula, we minimize the function

(6) $$min\frac{1}{2}\sum_{i=1}^{N}\sum_{j=1}^{N}\beta_{i}\beta_{j}x_{i}x_{j}+\varepsilon \sum_{i=1}^{N}\beta_{i}+\sum_{i=1}^{N}y_{i}\beta_{i}$$

with the constraints

(7) $$\sum_{n=1}^{N}\beta_{n}=0$$

So the parameter *w* can be described as a linear combination of the training observations,

(8) $$w=\sum_{n=1}^{N}\beta_{n}x_{n}$$

In this study, we used the LIBSVM toolbox developed by Chih-jen Lin to complete the following analysis [42].

### 2.7. Performance Analysis

Due to the limited number of samples, we used a leave-one-out cross-validation (LOOCV) strategy to estimate the generalization ability of the predictors. During LOOCV, each patient was designated as the test sample in turn, while the remaining patients were used to train the predictors. The performance of a predictor can be quantified using the sensitivity and classification accuracy based on the results of cross-validation. The two parameters are defined as follows:

Sensitivity is a ratio of the number of one anesthetic state, which is correctly identified as the total number of corresponding anesthetic states.

(9) $$S_{i}=\frac{N_{i,detected}}{N_{i,total}}$$

where *i* expresses four states (awake, light anesthesia, general anesthesia, deep anesthesia); $N_{i,detected}$ is the number of each correctly detected anesthetic state; $N_{i,total}$ is the number of each anesthetic state.

Classification accuracy is a ratio of the number of all anesthetic states which are correctly identified to the total number of actual anesthetic states.

(10) $$ACC=\frac{N_{detected}}{N_{total}}$$

where $N_{detected}$ is the number of all correctly detected anesthetic states; $N_{total}$ is the number of all anesthetic states.

In addition, the Pearson’s correlation coefficient between BIS and the index of the proposed method was also calculated to evaluate the proposed method. The Bland–Altman analysis was used to evaluate the agreement between the methods and the bias [43]. Finally, we compared the performance between the two classification methods, ANN and SVM. Due to our small sample size, our sample does not follow normal distribution. The nonparametric Wilcoxon signed-rank test does not require the data to follow normal distribution. Therefore, the Wilcoxon signed-rank test was used to compare the two classification methods

## 3. Results

**Figure 2:**
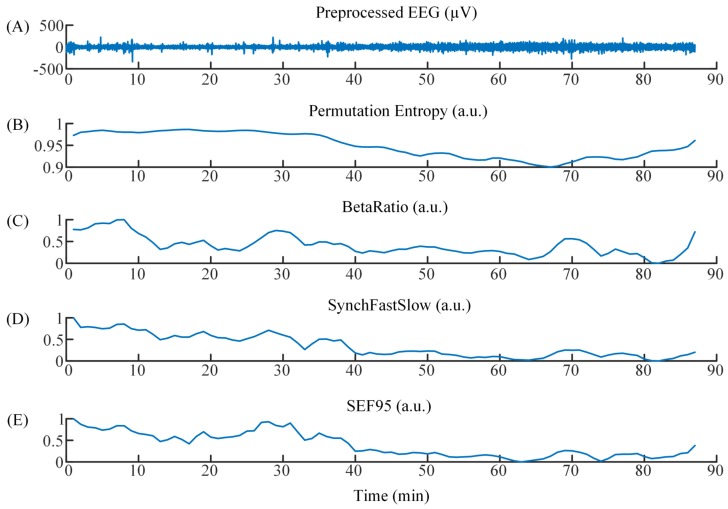
(**A**) The time course of preprocessed EEG. (**B**)–(**E**) The time courses of Permutation Entropy, BetaRatio, SynchFastSlow, and SEF95.

In this study, to be consistent with the output of BIS monitor (every 1 min), every 6 PE values were averaged (1 PE value needs data length of 10 s). The four features which were extracted from every 1-min EEG epoch were set as the inputs to the ANN to distinguish the awake, light, General, and deep anesthesia states. The time courses of preprocessed EEG and four features are shown in Figure 2.

**Figure 3:**
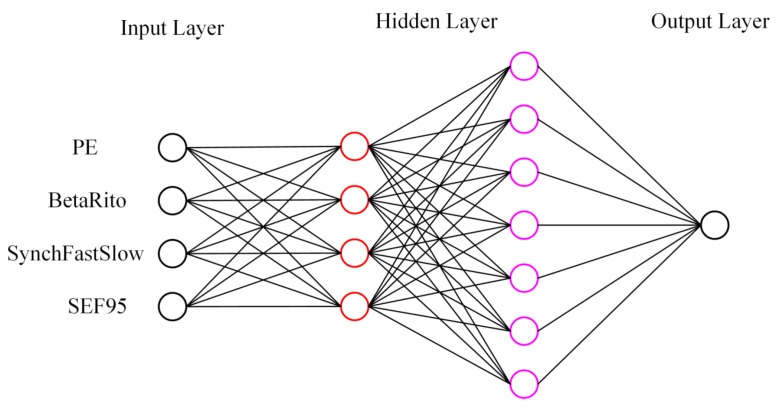
A schematic of the ANN structure used. One input layer with four nodes, the first hidden layer with four nodes, the second hidden layer with seven nodes, one output layer with one node.

To track the complex dynamics of EEG signal accurately, some different ANN structures were attempted according to the empirical formula of the number of hidden nodes: $d=\sqrt{a+b}+c$, where d is the number of hidden nodes. *a* and *b* are the number of input and output nodes, respectively. *c* is a regulation constant which ranges from 1 to 10. The ANN structure used in the current study consists of four layers: one input layer with four nodes, the first hidden layer with four nodes, the second hidden layer with seven nodes, one output layer with one node. The ANN structure is shown in Figure 3.

**Figure 4:**
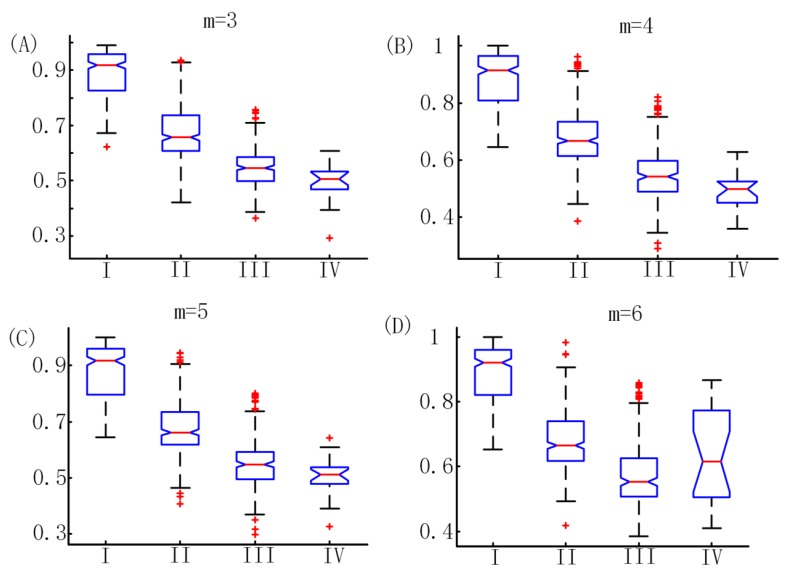
The distributions of PE values with m=3 to 6. I, II, III, and IV represent awake, light anesthesia, general anesthesia, and deep anesthesia, respectively. Vertical coordinates represent PE values.

To obtain the parameter m which made PE perform best in this study, we compared the performance of PE with m=3 to 6. The distributions of PE values with m=3 to 6 are shown in the box plots (Figure 4). It was obvious that PE could distinguish the awake, Light, and general anesthesia states, but exhibited a poor performance in detecting the deep anesthesia state with m=3 to 6. The deep anesthesia state identified by PE was confused with the general anesthesia state with m=3 to 5, and even light anesthesia state with m=6. The values of sensitivity and classification accuracy with different m values are listed in Table 2. It was found that the classification accuracy decreases with the embedding dimension m increasing. The best classification accuracy in monitoring DoA was 73.7% which was obtained with m=3. Obviously, a high sensitivity of 82.8% was obtained in detecting the awake state. However, the deep anesthesia state could not be identified well by the proposed method. The sensitivity of detecting the deep anesthesia state was only 8%. Thus, m=3 was used as the optimal parameter in the following analysis.

To demonstrate the superiority of the four features we selected in this study, we compared the performance (classification accuracies of all four anesthetic states) of all combinations of the features with ANN model. The detailed results are listed in Table 3. Obviously, the combination of the four features obtained the highest classification accuracy. This confirms that multiple features describing different anesthetic states can estimate the DoA better. In addition, we also compared the performance of ANN with SVM. The results are listed in Table 4. The ANN model yielded a higher classification accuracy of 79.1% (p=0.044, z=2.02). Meanwhile, the sensitivities of all four anesthetic states from ANN were higher than those from SVM.

**Figure 5:**
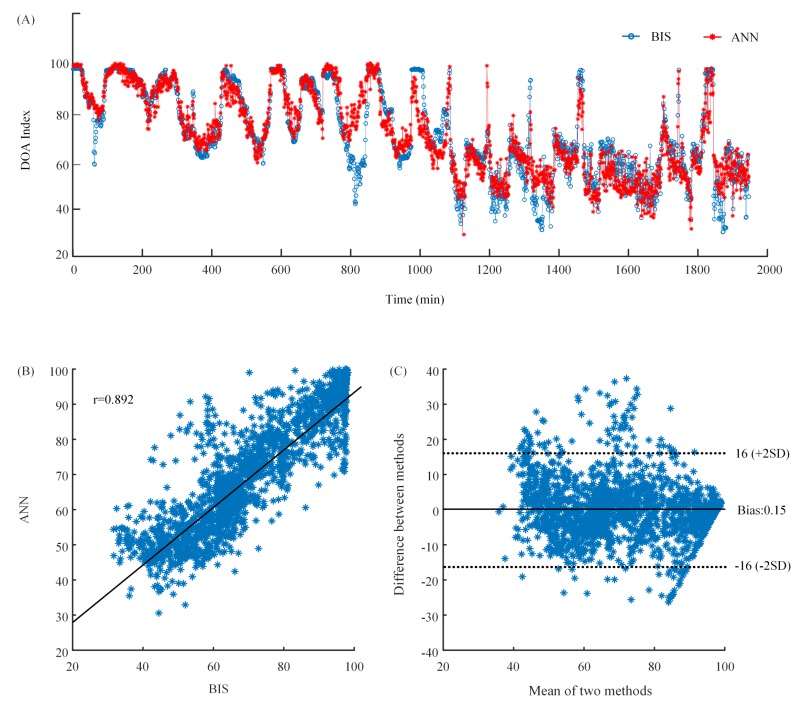
(**A**) The waveforms of BIS index and ANN output. The blue line is BIS index which is the target. The red line is ANN output which is the predicted value. (**B**) The scatter plot of BIS index and ANN output. (**C**) The Bland–Altman plot of BIS index and ANN output.

The results of cross-validation using four features are shown in Figure 5A. There was a high similarity between BIS and the index of the proposed method. In addition, the Pearson’s correlation coefficient between BIS and ANN outputs was 0.892 (Figure 5B). The bias calculated by the Bland–Altman analysis was 0.15. The limits of agreement were −16 and 16, indicating very little bias and a very good agreement (Figure 5C).

## 4. Discussion

DoA is a rather significant index for surgeons during surgery. In previous studies, some researchers have attempted to use the EEG-based features combined with ANN to assess DoA. However, they just used one EEG-based feature [2] or someone kind of EEG-based feature [4]. We think combining more EEG-based features with ANN may be better.

In this study, we proposed a method based on multiple EEG-based features, including frequency-domain feature and entropy feature, combined with ANN to assess the DoA. The datasets of 16 patients with propofol were used for evaluating the proposed method. A high classification accuracy was obtained in detecting awake, light, and general anesthesia states. However, the proposed method exhibited a poor performance in detecting the deep anesthesia state.

As described in previous studies, there are some reasonable causes that can explain these results. At low anesthetic concentrations, the frequency was in the beta range, but the frequency slowed down to approximately 8 Hz as the drug concentration increased. This state could be distinguished more accurately using the PE and BR. PE considers both the overall signal variability characteristics, which are naturally related to the spectral content, and the signal’s complexity or regularity [4]. In addition, PE is robust to the artifacts of eye movement and baseline drift during the awake state [23]. BR could well track the patient’s level of consciousness during the induction of anesthesia. At general and deep anesthesia states, SFS and SEF95 are dominant features, since SFS could reflect the frequency changes of EEG; meanwhile SEF95 reflects the degree of phase coupling [17]. However, the sample size of deep anesthesia state is too small in this study. In particular, the small number of deep anesthesia states caused poor performance in detecting the deep anesthesia state. Additionally, the performance of ANN with preprocessed EEG as input was not better than our strategy. The preprocessed EEG might contain too much redundant information, which affected the performance of ANN.

It should be noted that although we could well distinguish between awake and other anesthesia states, there are two limitations in this study. First, we did not consider the high variability in the human EEG due to our small sample size. Second, we did not test the drug variability, because the patients in this study were all anesthetized with propofol. These limits will be overcome in our future work.

## 5. Conclusions

We combined multiple EEG-based features, including frequency-domain feature and entropy feature, with ANN to assess the DoA. Our results showed that the proposed method could well distinguish between awake and other anesthesia states. The correlation coefficient between BIS and the index of the proposed method is generally high. This method used here is promising and feasible for a monitoring system to assess the DoA. In the future, we will increase the number of the patients and drug variability to continue to test our method.

## Figures and Tables

**Table 1:** The information of the EEG data.

Subject	Raw EEG Length (min)	Preprocessed EEG Length (min)	Number of Samples
Patient 1	139	130	130
Patient 2	139	138	138
Patient 3	170	168	168
Patient 4	135	134	134
Patient 5	88	87	87
Patient 6	68	63	63
Patient 7	134	129	129
Patient 8	129	126	126
Patient 9	110	109	109
Patient 10	108	108	108
Patient 11	126	125	125
Patient 12	138	137	137
Patient 13	168	168	168
Patient 14	124	124	124
Patient 15	88	80	80
Patient 16	124	121	121

**Table 2:** Sensitivity and classification accuracy of PE with different embedding dimension *m*.

	Sensitivity of	Sensitivity of	Sensitivity of	Sensitivity of	Classification
	Awake	Light Anesthesia	General Anesthesia	Deep Anesthesia	Accuracy
m=3	82.8%	65.5%	81.3%	8%	73.7%
m=4	81.5%	64.2%	80.3%	4%	72.4%
m=5	80.7%	63.6%	79.4%	6%	71.8%
m=6	79.8%	60.6%	81.5%	2%	70.7%

**Table 3:** The results of all combinations of 1, 2, 3, 4 features with ANN.

Single	Classification	Two	Classification	Three	Classification	Four	Classification
Feature	Accuracy	Features	Accuracy	Features	Accuracy	Features	Accuracy
PE	73.7%	PE-SFS	75.7%	PE-SFS-BR	76.2%	PE-SFS-BR-SEF95	79.1%
SFS	63.6%	PE-BR	76.0%	PE-SFS-SEF95	76.8%		
BR	60.4%	PE-SEF95	75.5%	PE-BR-SEF95	75.8%		
SEF95	66.7%	SFS-BR	64.6%	SFS-BR-SEF95	71.8%		
		SFS-SEF95	69.1%				
		BR-SEF95	64.4%				

**Table 4:** Comparison between ANN and SVM with four features.

	Sensitivity of	Sensitivity of	Sensitivity of	Sensitivity of	Classification
	Awake	Light Anesthesia	General Anesthesia	Deep Anesthesia	Accuracy
ANN	86.4%	73.6%	84.4%	14%	79.1%
SVM	84.8%	71.1%	82.1%	2%	76.7%

**Figure A1:**
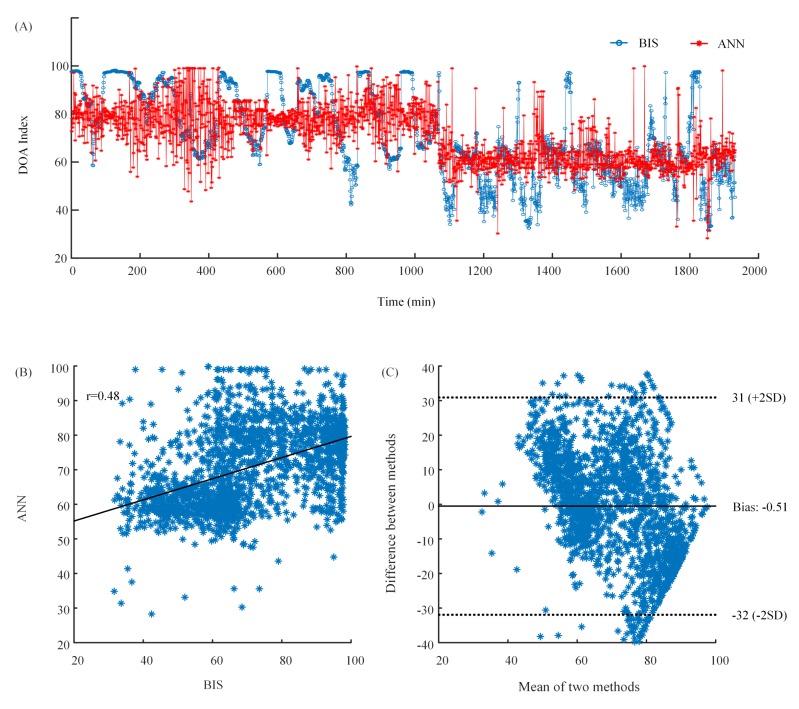
(**A**) The waveforms of BIS index and ANN output. The blue line is BIS index which is the target. The red line is ANN output which is the predicted value. (**B**) The scatter plot of BIS index and ANN output. (**C**) The Bland–Altman plot of BIS index and ANN output.

## Appendix A

Considering that ANN might work better with raw signals as input, we used the preprocessed EEG as input to train the ANN. Then we used the same LOOCV strategy and performance metrics to estimate the generalization ability of the ANN. However, we found the results were not better. The behavior of BIS and ANN outputs are shown in Figure A1. The Pearson’s correlation coefficient between BIS and ANN outputs was only 0.48. The classification accuracy of all four states of anesthesia was only 42.2%. The bias calculated by the Bland–Altman analysis was −0.51. The limits of agreement were −31 and 31. The sensitivity and classification of this strategy are listed in Table A1.

**Table A1:** The results of ANN with preprocessed EEG as input.

	Sensitivity of	Sensitivity of	Sensitivity of	Sensitivity of	Classification
	Awake	Light Anesthesia	General Anesthesia	Deep Anesthesia	Accuracy
ANN	36.2%	51.4%	39.7%	6%	42.2%
